# Technical accuracy of optical and the electromagnetic tracking systems

**DOI:** 10.1186/2193-1801-2-90

**Published:** 2013-03-08

**Authors:** Tapani Koivukangas, Jani PA Katisko, John P Koivukangas

**Affiliations:** 1 Department of Mechanical Engineering, University of Oulu, PL 4200, Oulu, 90014 Finland; 2 Department of Neurosurgery, Oulu University Hospital, Oulu, 90029 Finland; 3 Department of Neurosurgery, University of Oulu, Oulu, 90029 Finland

**Keywords:** Computer assisted surgery, Optical tracking system, Electromagnetic tracking system, Technical accuracy, Region of surgical interest

## Abstract

Thousands of operations are annually guided with computer assisted surgery (CAS) technologies. As the use of these devices is rapidly increasing, the reliability of the devices becomes ever more critical. The problem of accuracy assessment of the devices has thus become relevant. During the past five years, over 200 hazardous situations have been documented in the MAUDE database during operations using these devices in the field of neurosurgery alone. Had the accuracy of these devices been periodically assessed pre-operatively, many of them might have been prevented.

The technical accuracy of a commercial navigator enabling the use of both optical (OTS) and electromagnetic (EMTS) tracking systems was assessed in the hospital setting using accuracy assessment tools and methods developed by the authors of this paper. The technical accuracy was obtained by comparing the positions of the navigated tool tip with the phantom accuracy assessment points. Each assessment contained a total of 51 points and a region of surgical interest (ROSI) volume of 120x120x100 mm roughly mimicking the size of the human head.

The error analysis provided a comprehensive understanding of the trend of accuracy of the surgical navigator modalities. This study showed that the technical accuracies of OTS and EMTS over the pre-determined ROSI were nearly equal. However, the placement of the particular modality hardware needs to be optimized for the surgical procedure. New applications of EMTS, which does not require rigid immobilization of the surgical area, are suggested.

## Introduction

Current operating rooms are equipped with complex devices and machines. The most critical operations in minimally invasive surgery rely largely on the seamless co-operation between these technologies and the surgeons. The goal is to treat the patient with the best possible outcome using the most suitable method. The more operations are performed using image guided surgical devices, i.e. surgical robots and navigators, the higher the requirement becomes for accuracy and reliability. Surgical guidance device technologies improve the operational quality by guiding the used instrument with sub-millimetric accuracy inside the human body with a virtual view for the surgeon on a computer screen.

The use of surgical guidance devices has led to computer assisted surgery (CAS). The main advantage reached with CAS is that the surgical procedure can be planned preoperatively with images taken from the patient and then performed using surgical navigators for instrument guidance (Wiles et al. 2004; Beaulieu et al. 2008; Grunert et al. 2003; Kücker et al. 2006; Mascott 2005). This is made possible by spatially linking the patient and the surgical instrument to the image data (Kücker et al. 2006). Two main modalities of navigator tracking have been adopted, namely the optical (OTS) and electromagnetic (EMTS) tracking systems.

The main disadvantage of the OTS is the need for a clear line-of-sight between the patient and instrument trackers and the optical cameras. This limits the use of the OTS in some CAS procedures (Chung et al. 2004; Katisko 2012; Schneider & Stevens 2011; Birkenfeller et al. 1998). The wireless capability of passive OTS and lightness of weight together with linearity, stability and accuracy are the clear advantages of the system (Katisko 2012; Birkenfeller et al. 1998; Vahala et al. 2001; Simon 1997). Possible causes of the decrease in the accuracy of OTS include camera lens and image distortions and rough handling (Wiles et al. 2004).

The EMTS has been reported to encounter problems when metallic objects are in the presence of the operated area (Birkenfeller et al. 1998; Frantz et al. 2003; Schicho et al. 2005). This has somewhat hampered the widespread use of EMTS in surgical procedures. Also the need for a wired connection of the trackers and the EM field generator to the module box further limits the wider adoption of this tracking modality (Chung et al. 2004; Katisko 2012; Schneider & Stevens 2011; Birkenfeller et al. 1998). The greatest advantage reached with EMTS is that it does not require a clear line-of-sight between the field generator and the sensors (Mascott 2005; Chung et al. 2004; Frantz et al. 2003). Another advantage over the OTS is that since the sensing coils are close to the tip of the tracked instrument, and thus closer to the point of surgical interest, tracking inside the human body with a flexible instrument is possible. Recently, miniaturization of the sensor coils has lowered the effect on the accuracy of other metals in the presence of the field generator (Birkenfeller et al. 1998).

As the use of surgical guidance devices has seen an increase in minimally invasive surgery (MIS) (Howe & Matsuoka 1999; Zoppi & Khan 2010; Stiehl et al. 2007), the need for the accuracy requirements of the manipulators has also been realized. International organizations such as the American Society for Testing and Materials (ASTM) and other international organizations are making efforts to standardize medical robotics accuracy assessment (Howe & Matsuoka 1999; Zoppi & Khan 2010; Stiehl et al. 2007; Haidegger et al. 2010; Kroneif et al. 2012). Yet, there are no widely accepted methods or regulations.

The present study at Oulu University Hospital concentrated on the technical accuracy assessment of the OTS and the EMTS modalities in a way that enables periodic assessment in the hospital setting. The accuracy of a commercial navigator enabling the use of both modalities was assessed in the region of neurosurgical interest. The tools and methods for this research have been developed by the authors of this paper to improve the safety of CAS procedures and thus the quality of operations and patient treatment. The goal of this study was also to compare the trends of error of the two modalities and thus further evaluate their possible applications.

## Research equipment and methods

This study used a specially designed accuracy assessment phantom (Koivukangas 2012) to assess the accuracy of a commercial surgical navigator, the StealthStation S7 (Medtronic Inc., Louisville, CO, USA). The navigator enabled the interchangeable use of both OTS and EMTS. The phantom consisted of three separate levels attached with screws to form the total reference volume. On each level a total of 49 accuracy assessment points were machined with 20 mm displacement between the beveled holes. This gave a specific region of surgical interest (ROSI) volume of 120x120x100 mm. The phantom was industrially verified at Oulu PMC using the Mitutoyo Strato 9166 (Mitutoyo, Japan) accuracy sensing device. The displacement error of the accuracy assessment points was found to be +/− 0.015 mm.

The tests were conducted at Oulu University Hospital in a space adjacent to the operating rooms. The accuracy assessment of both tracking modalities was done in a sequence of five experiments. Each experiment set was done with 17 chosen accuracy assessment points on each of the three levels for a total of 51 points. The optical camera and the patient tracker of the navigator were placed in a typical OTS surgical configuration as indicated by the navigator software. Thus, the distance from the optical camera pair to the center of the phantom was 1.90 m. The magnetic field generator and the patient tracker were placed in the same manner for the EMTS analysis.

The objective of this study was not only to compare the accuracy of OTS versus EMTS, but also to prove that the materials and methods could be used in the hospital setting for periodic assessment of accuracy. At the time of testing, the Stealth Station S7 had been in routine surgical use for three years at the hospital.

**Figure 1:**
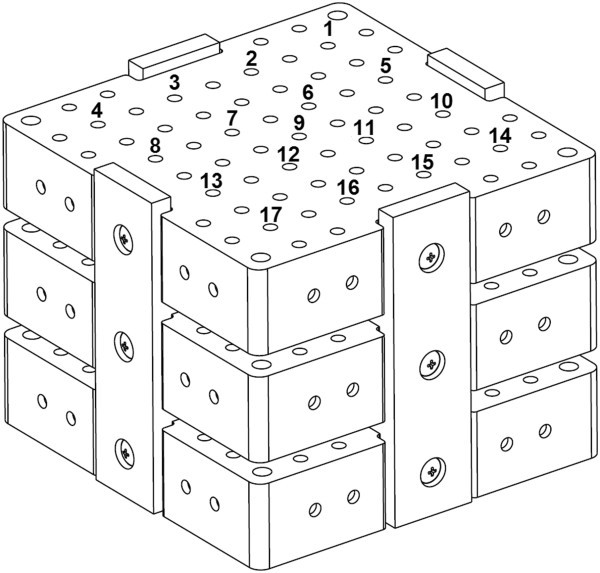
**A 3D CAD image of the designed accuracy assessment phantom, with the numbers corresponding to the accuracy assessment points used for each level.**

The accuracy assessment phantom was fixed on a measurement platform. The accuracy data was collected using both tracking modalities. The accuracy assessment protocol was based on collecting the coordinate data in X, Y and Z directions of each accuracy assessment point from point 1 to point 17 on each level of the phantom. The accuracy assessment phantom and protocol are illustrated in Figure 1*.* The center point on the middle level (point 9) was standardized as the reference point of origin by comparing the coordinates of the other coordinate points to those of this point.

The error at each accuracy assessment point was calculated using (1), (Koivukangas 2012):1

where *X*_*ij*_, *Y*_*ij*_ and *Z*_*ij*_ are the ‘true’ values on the phantom and *X*_*M,ij*_*, Y*_*M,ij*_ and *Z*_*M,ij*_ are the *i-th* accuracy assessment point coordinates measured on the phantom and *j* (Wiles et al. corresponds to the analyzed level on the phantom*, i ∈ [1,49], j ∈*^2004^; Grunert et al. 2003)*. 25* corresponds to the middle accuracy assessment point.

The mean error, *E*_*MEAN*_, was obtained using (2), the RMS error, *∈*, using (3) and the 95% confidence interval using (4):2

3

4

where *E*_*ij*_ indicates the mean error at each corresponding accuracy assessment point gained using (1), *n* indicates the number of assessed points and *σ* indicates standard deviation.

## Results

**Figure 2:**
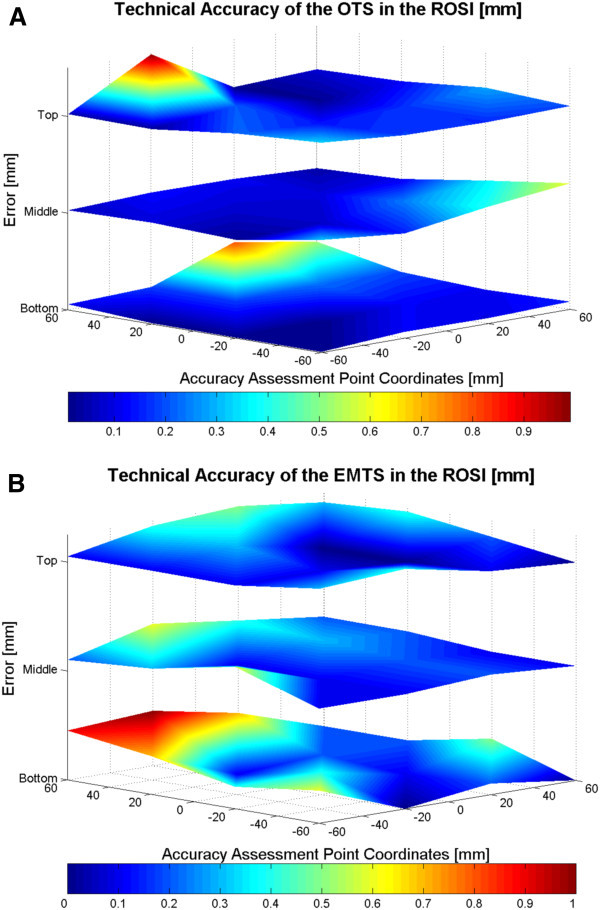
**The 3D error surfaces of the OTS (A) and the EMTS (B).** The trend of error for both modalities is seen with the changes of the shading within the surfaces. The dark blue color represents 0 mm error and dark red the highest error. The orientation of the phantom with respect to the navigator is such that the optical camera pair of the OTS (**A**) is located perpendicularly to the right front edge and the EM field generator of the EMTS (**B**) perpendicularly to the right front edge, in both cases at the middle level.

**Figure 3:**
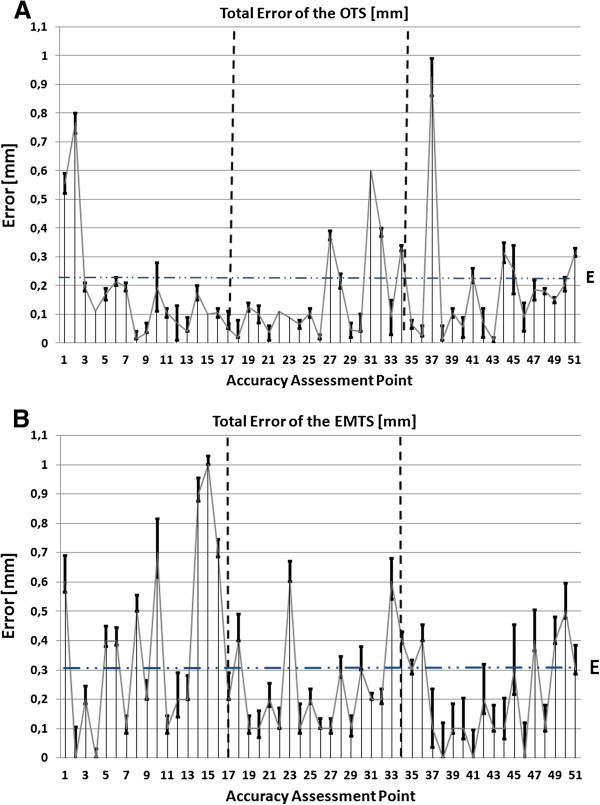
**A sequence plot representation of the errors for the OTS (A) and the EMTS (B).** The first plot on each graph represents the first accuracy assessment point (point 1 on the bottom level) and the last plot the 51^st^ point (the last point on the top level) of the phantom. The vertical bars represent the error interval at each point.

**Figure 4:**
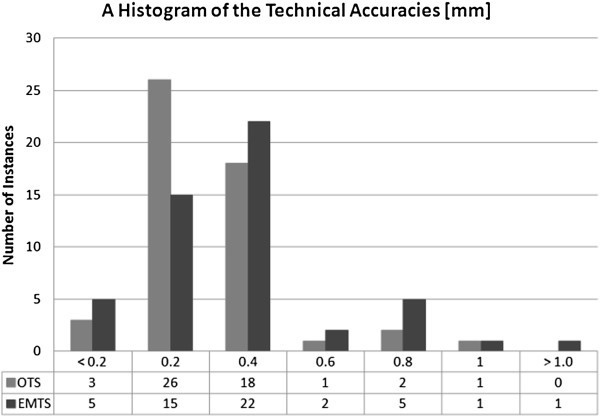
**A histogram of the accuracy of the tracking modalities.**

The results for the accuracy of the surgical navigator modalities are presented as follows. First, a 3D error surface representation shows the trend of the error over each accuracy assessment level of the ROSI (Figure 2). Then, a sequence plot diagram shows the error in detail at each corresponding point (Figure 3). The histogram (Figure 4) shows the error as numbers of instances at 0.20 mm increments. Table 1 collects the data for quick comparison of each tracking modality.

**Table 1:** **The technical accuracy analysis of the OTS and the EMTS**

Error [mm]		
	**OTS**	**EMTS**
Mean Error, *EMEAN*	0.20	0.30
RMS Error, ϵ	0.27	0.36
95% CI	0.60	0.76
Standard Deviation, σ	±0.10	±0.13
Error, *EMAX*	0.99	1.10

Figure 2 illustrates the error of each tracking system on 3D error surfaces. The trend of the errors and the errors in millimeters at each corresponding accuracy assessment point can be seen. The Figures show that the error trends differ slightly between the modalities. For the OTS (Figure 2A) the dependence of the error on the distance from the optical camera to the object can be seen, as the error is clearly skewed to the furthest edge of the ROSI volume. There is a slight error increase also close to the patient tracker on the middle level of the phantom. For the EMTS (Figure 2B) the dependence of the error on the distance from the electromagnetic (EM) field generator to the object is evident, as the error is slightly skewed at the closest proximity to the EM field generator and highly skewed at the furthest point from the generator. The distance from the field generator to the furthest point on the phantom in the experiments was approximately 35 cm.

Each level on the accuracy assessment phantom contained 17 accuracy assessment points. The trend of the error can thus also be seen in the sequence diagrams (Figure 3). The error distributions in the three levels correspond to those in Figure 2, but are unique for both tracking modalities with less deviation being seen with the OTS.

Figure 4 contains the accuracy data in a histogram. The histogram shows the distribution of the error of each modality within each fifth of a millimeter. The histogram together with the 95% CI can be used as a tool for showing the dependence of the outlier errors with respect to the technical accuracy. As the errors are represented as distance values, which by definition are presented as positive numbers, the 95% CI clarifies the accuracy of the tested device and strengthens the technical accuracy results by showing the user the effect of highest errors on the total accuracy.

Table 1 combines the pertinent aspects of the error analysis. Overall results for the OTS and the EMTS show mean technical accuracies of 0.20 mm ± 0.10 mm and 0.30 mm ± 0.13 mm, respectively.

## Discussions and conclusions

This paper compares in a hospital setting the technical accuracies of the two most widely used navigator tracking modalities using the same accuracy assessment tools and methods within the same ROSI. The study shows little difference in the accuracies of the OTS and EMTS but that the placement of the tracking hardware needs to be optimized for surgical procedures. The results also indicate that acceptable accuracy had been maintained after three years of routine surgical use.

The technical accuracy of the OTS was found to be 0.20 mm ± 0.10 mm. Other international groups assessing the OTS have reported technical accuracies in the range of 0.1 mm to 1.4 mm, depending on the assessed device and methods (Wiles et al. 2004; Kücker et al. 2006; Mascott 2005; Simon 1997; Alakuijala 2001; NDI Digital 2012; Ringel et al. 2009; Ruohonen & Karhu 2010; Widmann et al. 2012; Wittmann et al. 2011). NDI, the manufacturer of optical cameras commonly used in surgical navigators, reports an accuracy of 0.25 mm with a 95% CI of 0.5 mm for their new products, the Polaris Spectra^®^ and Polaris Vicra^®^ (NDI Digital 2012).

This study further shows the dependence of the error on the distance from the optical camera to the object, as the error was clearly skewed to the furthest edge of the ROSI volume. There was also a slight error increase close to the patient tracker on the middle level of the phantom. This phenomenon can be seen as a systematic error in the 3D representation and the error sequence plot diagram. A possible reason for this is that optical cameras have been shown to contain possible lens distortion when using them as tracking methods (Schneberger 2004; Weng et al. 1992). Schneberger has also shown the possible influence of different factors and components of OTS on technical accuracy: 2D calculation of the patterns accounting for 0.02 mm, residual image distortions for up to 0.17 mm, thermal drift for up to 0.11 mm, scaling for 0.26 mm and noise for an additional 0.01 mm, summing up to 0.47 mm error (Schneberger 2004).

The technical accuracy of the EMTS was 0.30 mm ± 0.13 mm. The EMTS technical accuracy was earlier found to be in the range of 0.17 mm to 1.4 mm (Mascott 2005; Schneider & Stevens 2011; Simon 1997; Frantz et al. 2003; Schicho et al. 2005; Alakuijala 2001; de Lambert et al. 2012; Hummel et al. 2006). From these results it is evident that EMTS is a slightly more error sensitive method. For the EMTS the dependence of the error on the distance from the electromagnetic (EM) field generator to the object was evident, as the error was slightly skewed at the closest proximity to the EM field generator and highly skewed at the furthest point from the generator as shown in the 3D representation and the error sequence plot diagram.

After all, as the difference between the technical accuracies of the OTS and EMTS was found to be marginal, the most suitable method of instrument tracking for surgical procedures can be chosen using criteria other than purely technical accuracy. The greatest difference between the tracking modalities involves the tracking of the instruments used. Compared with the OTS, the EMTS has unique advantages for special surgical procedures. The tracked coils are placed near the end of the tip of the EMTS guided instrument, while the reflecting spheres or active markers on the OTS need to be placed farther away to provide line-of-sight visibility (Verbakel et al. 2010). This significant difference between the tracking methods means that the EMTS instrument tip is tracked closer to the sensitive anatomical structures. Also, the OTS requires stable positioning and immobilization of the patient (Koivukangas 2012). Since the EMTS patient trackers are light and may be attached to the patient without for example skull clamps, rigid immobilization of the patient during procedures is not as critical. Furthermore, EMTS makes navigation possible even for infants as immobilization is not necessarily required as long as the patient tracker is securely attached to the patient. On the other hand, some EMTS instruments are quite flexible and if subjected to a bending force, the method may result in significant accuracy errors. Since EMTS navigation is based on tracking the coils of the instrument, the relationship between the coils must not be changed during the procedure.

Thousands of operations are annually guided using CAS technologies. However, as the use of these devices is rapidly increasing, the reliability of the devices becomes ever more critical. A problem for accuracy assessment of the devices has thus become evident. By exploring the MAUDE (MAUDE 2012) database under the FDA, over 200 hazardous situations have been documented during operations that led to injury and longer hospital stay using CAS devices in the field of neurosurgery alone. Had the accuracy of these devices been periodically assessed pre-operatively in the hospital setting, probably a number of them could have been prevented.

## Abbreviations

3D: Three-dimensional
ASTM: American Society for Testing and Materials
CAD: Computer aided design
CAS: Computer assisted surgery
EM: Electromagnetic
EMTS: Electromagnetic tracking system
FDA: Food and Drug Administration
MAUDE: Manufacturer and User Facility Experience Database an online database of the FDA
MIS: Minimally invasive surgery
OTS: Optical tracking system
ROSI: Region of surgical interest
